# Statistical Genetics and Its Applications in Medical Studies

**DOI:** 10.1155/2014/712073

**Published:** 2014-03-06

**Authors:** Ao Yuan, Wenqing He, Gengsheng Qin, Qizhai Li

**Affiliations:** ^1^Department of Biostatistics, Bioinformatics and Biomathematics, Georgetown University, Washington, DC 20057, USA; ^2^Department of Statistics and Actuarial Science, University of Western ontario, London, ON, Canada N6A 5B7; ^3^Department of Mathematics and Statistics, Georgia State University, Atlanta, GA 30303, USA; ^4^Academy of Mathematics and Systems Science, Chinese Academy of Sciences, Beijing 100190, China

Statistical genetics can be viewed as a classical branch of applied probability and statistics, which has recently gained much momentum, due to the significant breakthroughs in genetics. With the availability of modern techniques, new methods, and significantly increased data information, it is imperative to study the relationship between gene traits such as diseases and genetic susceptibilities in an unprecedented manner. This area is among the hottest topics in applied statistics, applied mathematics, biological/medical studies, and other related sciences.

The main aim of this special issue focuses on the new development and applications of computational, mathematical, and statistical methods in genetic disease study. The special issue could become an international forum for researchers to exchange their new thoughts, most recent developments, and ideas in the field.

In this special issue, we selected seven articles within the above topics. Below is a brief summary of the selected articles in this special issue.

Recent advances in biotechnologies have led to the identification of an enormous number of genetic markers in disease association studies; how to select a smaller set of genes to explore the relation between genes and disease is a challenging task. Bayesian methods have the advantage of incorporating prior information into the model for such analysis. Article “*Applications of bayesian gene selection and classification with mixtures of generalized singular g-priors*” by W.-K. Chien and C. K. Hsiao addresses this problem using Bayesian method with a Gaussian prior and inverse gamma hyperprior. The proposed approach is applied to a colon and leukemia cancer study. Comparison with other existing methods was conducted. The authors find that classification accuracy of the proposed model is higher with a smaller set of selected genes and that the results not only replicated findings in several earlier studies, but also provided the strength of association with posterior probabilities.

Article “*Modified logistic regression models using gene coexpression and clinical features to predict prostate cancer progression*” by H. Zhao et al. proposed a new logistic regression model for predicting prostate cancer progression. They incorporated coexpressed gene profiles into the logistic model based on clinical features to improve the inference accuracy. Then they use the top-scoring pair method to select genes with significant association with the disease. The performance of the proposed method is compared with some commonly used methods for such problem, using data sets from such published studies. Their study suggests that the proposed method performs better than a commonly used one and that the top-scoring pair method is a useful tool for feature (and/or gene) selection to be used in prognostic models.

Resampling-based multiple testing procedures are widely used in genomic studies to identify differentially expressed genes and for genome-wide association studies. The power and stability of these popular procedures have not been extensively evaluated. Article “*Power and stability properties of resampling-based multiple testing procedures with applications to gene oncology studies*” by D. Li and T. D. Dye investigates the power and stability of seven commonly used resampling-based multiple testing procedures that are frequently used in high-throughput data analysis for small sample size data. Simulations and real data gene oncology examples are employed in their investigation. Their study suggests that the bootstrap single-step minP procedure and the bootstrap step-down minP procedure perform the best, when sample size is as small as 3 in each group and either familywise error rate or false discovery rate control is desired. When sample size increases to 12 and false discovery rate control is desired, the permutation maxT procedure and the permutation minP procedure perform the best.

Article “*Transcriptional protein-protein cooperativity in POU/HMG/DNA complexes revealed by normal mode analysis*” by D. D. Wang and H. Yan investigates how proteins in POU/HMG/DNA ternary complexes interact cooperatively, which are crucial in transcriptional regulation of embryonic stem cells. They use the normal mode analysis to detect the most cooperative or collective motions (essential modes) of a large number of proteins, a commonly used tool to analyze the structural dynamics of biomolecules, which combines some techniques in engineering, mathematics, and statistics. Their work reveals how the two proteins Oct-1 and Sox-2 work together physically and structurally at two specific DNA biding sites, by analyzing the motion magnitude functions. A correlation measure is used to characterize the amount of cooperativity of pairs of proteins. The proposed methods provide useful information for understanding the complicated interaction mechanism in the POU/HMG/DNA complexes. The corresponding online computational tools are also provided.

In modern medical diagnosis or genetic studies, the receiver operating characteristic (ROC) curve is a popular tool to evaluate the discrimination performance of biomarkers on a disease status or a phenotype. With the presence of a number of covariates in the data, how to select the most relevant covariables, or to select the model with good overall properties, is a challenging problem. Article “*Variable selection in ROC regression*” by B. Wang addresses this problem with an interesting idea. There are a large number of criteria available for this problem. The author first rewrites the ROC regression into a grouped variable selection form so that current criteria can be applied and then proposes a general two-stage framework with a BIC selector for the group SCAD algorithm under the local model assumption. Basic asymptotic properties of the proposed methods are derived. Simulation studies and real data analysis show that the proposed grouped variable selection is superior to the traditional model selections. Furthermore, the author finds that the focused information criterion provides more accurate estimated area under the ROC curve compared with other criteria.

Two-stage design and analysis are often adopted in genome-wide association studies (GWASs). Considering the genetic model uncertainty, many robust procedures have been proposed and applied in GWASs. The existing approaches mostly focused on binary traits, and many of these methods analyze data based on two separate stages, and few work has been done on continuous (quantitative) traits. Article “*Robust joint analysis with data fusion in two-stage quantitative trait genome-wide association studies*” by D.-D. Pan et al. proposes a powerful F-statistic-based robust joint analysis method for quantitative traits using the combined raw data from both stages, in which the genetic effects are modeled as regression parameters. Variations of the MAX testing statistic are constructed to calculate the statistical significance and power. It is well known that critical values and power of the MAX type statistic are not easy to compute. The authors derived analytic expressions on the basis of the asymptotic distributions, so that these quantities can be easily obtained. They show using simulations that the proposed method is substantially more robust than the *F*-test based on the commonly used additive model when the underlying genetic model is unknown.

Multiple meta-analyses may use similar search criteria and focus on the same topic of interest, but they may yield different or sometimes discordant results. The lack of statistical methods for synthesizing these findings makes it challenging to properly interpret the results from multiple meta-analyses, especially when their results are conflicting. Article “*A statistical method for synthesizing meta-analyses*” by L. L. Tang et al. introduces a method to synthesize the meta-analytic results under two cases: (1) when multiple meta-analyses use the same type of summary effect estimates and (2) when meta-analyses use different types of effect sizes. In case 2, the meta-analysis results cannot be directly combined; therefore they propose a two-step frequentist procedure to first convert the effect size estimates to the same metric and then summarize them with a weighted mean estimate. The proposed method has the following advantages over some existing methods: different types of summary effect sizes can be considered; the same overall effect size can be provided by conducting a meta-analysis on all individual studies from multiple meta-analyses.

One of the main objectives of a genome-wide association study (GWAS) is to develop a prediction model for a binary clinical outcome using single-nucleotide polymorphisms (SNPs) which can be used for diagnostic and prognostic purposes and for better understanding of the relationship between the disease and SNPs. Penalized support vector machine (SVM) methods have been widely used toward this end. However, since investigators often ignore the genetic models of SNPs, a final model results in a loss of efficiency in prediction of the clinical outcome. Article “*SNP selection in genome-wide association studies via penalized support vector machine with MAX test*” by J. Kim et al. proposes a two-stage method such that the genetic models of each SNP are identified using the MAX test and then a prediction model is fitted using a penalized SVM method. They apply the proposed method to various penalized SVMs and compare their performances using various penalty functions. They show by simulations and real GWAS data analysis that the proposed method performs better than the prediction methods that ignore the genetic models, in terms of prediction power and selectivity.

Using DNA sequence data in the study of ancestral history of human population is an essential part in the understanding of human evolution. The existing methods for such coalescence inference using the method of either the rooted tree or unrooted tree constructed from the observed data, both of which use recursion formulae to compute the data probabilities. These methods are useful in practical applications but computationally complicated. Article “*On coalescence analysis using genealogy rooted trees*” by A. Yuan et al. explores a new method for this problem. They first investigate the asymptotic behavior of such inference; their results indicate that, broadly, the estimated coalescent time will be consistent to a finite limit. Then they study a relatively simple computation method for this analysis and illustrate how to use it.

